# The Associations between Erythropoietic Response with Inflammation Markers and Perfluorinated Chemicals in Hemodialysis Patients

**DOI:** 10.3390/healthcare11030442

**Published:** 2023-02-03

**Authors:** Wen-Sheng Liu, Chien-Hung Lin, Ann Charis Tan, Yen-Ting Lai, Tsung-Yun Liu, Hsiang-Lin Chan, Szu-Yuan Li, Chun-Fan Chen, Yung-Tai Chen, Tz-Heng Chen, Fan-Yu Chen, Yang Ho, Han-Hsing Tsou, Chih-Ching Lin

**Affiliations:** 1 Division of Nephrology, Department of Medicine, Taipei City Hospital, Zhongxing Branch, Taipei 103, Taiwan; 2 School of Medicine, National Yang Ming Chiao Tung University, Hsinchu 300, Taiwan; 3 Institute of Food Safety and Health Risk Assessment, National Yang Ming Chiao Tung University, Hsinchu 300, Taiwan; 4 College of Science and Engineering, Fu Jen Catholic University, New Taipei City 242, Taiwan; 5 Department of Special Education, University of Taipei, Taipei 100, Taiwan; 6 Department of Pediatrics, Taipei Veterans General Hospital, Taipei 112, Taiwan; 7 Division of Nephrology, Department of Medicine, Taipei Veterans General Hospital, Taipei 112, Taiwan; 8 Department of Physical Medicine and Rehabilitation, National Taiwan University Hospital Hsin-Chu Branch, Hsinchu 300, Taiwan; 9 Department of Child Psychiatry, Linkou Chang Gung Memorial Hospital and University, Taoyuan 333, Taiwan; 10 Department of Internal Medicine, National Yang Ming Chiao Tung University Hospital, Yilan 260, Taiwan; 11 Division of Nephrology, Department of Internal Medicine, Taipei City Hospital, Heping Fuyou Branch, Taipei 100, Taiwan; 12 Division of Nephrology, Department of Medicine, Taipei Veterans, General Hospital Yuli Branch, Hualien 981, Taiwan

**Keywords:** continuous erythropoietin receptor activator, erythropoiesis-stimulating agents, hemodialysis, inflammation, erythropoietic response, perfluorinated chemicals

## Abstract

Erythropoiesis-stimulating agents (ESA) are used to treat anemia in hemodialysis (HD) patients. We investigated the role of inflammation and accumulation of environmental toxins (perfluorinated chemicals (PFCs), such as perfluorooctanoic acid and perfluorooctane sulfonate) in the erythropoietic response of HD patients who receive a fixed monthly continuous erythropoietin receptor activator (CERA) dosage. Forty-five patients underwent three successive phases of ESA treatment for two months each (phase one: 100 µg CERA once monthly; phase two: 50 µg CERA twice monthly; phase three: 100 µg CERA once monthly). Patient data were collected to determine the association of various factors with erythropoietic response (change in hematocrit). Liquid chromatography-tandem mass spectrometry was used to analyze perfluorinated chemicals. Twenty-eight patients exhibited a poor erythropoietic response that was significantly associated with: age > 80 years, initial hematocrit > 36%, glucose > 200 mg/dL, alanine aminotransferase > 21 U/L, c-reactive protein > 1 mg/dL, interleukin−6 > 10 ng/mL, lactate dehydrogenase ≤ 190 U/L, and chloride ≤ 93 mEq/L. There was also a borderline significant association between inflammation and PFCs, although PFCs failed to show any impact on ESA response. Age, glucose, chloride, liver function, and inflammation may be associated with cost-effective fixed CERA dosage administered at an increased frequency.

## 1. Introduction

Taiwan has the highest incidence and prevalence of end-stage renal disease (ESRD) among all developing countries [1]. The country’s National Health Insurance system provides full reimbursements for dialysis treatments, equating to an almost completely free dialysis system, with approximately 90% of uremic patients under hemodialysis (HD) [2]. One of the frequent complications of ESRD is anemia, due to erythropoietin deficiency. Erythropoiesis-stimulating agents (ESA) are often used for treating anemia in patients [3]. Several factors that influence ESA hyporesponsiveness resulting in the need for higher ESA dosage include iron deficiency, low serum albumin level, elevated aluminum level, chronic hyperparathyroidism, malnutrition, cardiovascular medications, inflammation, and suboptimal dialysis, that may eventually lead to the accumulation of uremic toxins [4,5,6,7].

The problem of environmental pollution is worsening in most developing countries. Persistent organic pollutants are strong lipophilic chemicals with long half-lives that have been associated with immune, nervous, and reproductive system diseases, because of their ability to bioaccumulate and biomagnify [8,9]. Perfluorinated chemicals (PFC) are pollutants widely used as surfactants. The most commonly used PFCs are perfluorooctanoic acid (PFOA) and perfluorooctane sulfonate (PFOS) [10]. Previous studies have demonstrated that the accumulation of these environmental toxins in the body leads to adverse effects that include decreased sex hormone levels, impaired glucose homeostasis, metabolic syndrome, and abnormal thyroid function [11,12,13].

Due to the long half-lives of PFCs and the harmful effects they may exert on the human body, continuous erythropoietin receptor activator (CERA, specifically Mircera), a long-acting ESA, (half-life of 139 h, licensed for once-a-month dosing) was chosen for this study [14]. A previous study showed that a change from darbepoetin-alpha (a shorter-acting ESA compared to CERA) to CERA may alleviate inflammation and decrease serum aluminum [15]. A higher LDL level is a proven predictor of poor ESA response [15], while PFOS is related to higher LDL and elevated liver function [12].

This study aims to investigate the role of inflammation and environmental toxin accumulation, and evaluate the erythropoietic response in ESRD patients under HD who receive a fixed CERA dosage with different frequencies of administration.

## 2. Materials and Methods

### 2.1. Inclusion and Exclusion Criteria

This is a prospective study approved by the institutional review boards of Taipei Veterans General Hospital (IRB 2011-10-004IA) and Taipei City Hospital (TCHIRB-10901017-E). Study recruitment was conducted at the nephrology departments of the aforementioned hospitals. Patients who were stable and received CERA as ESA treatment were enrolled in the study to evaluate their response after different dosage intervals. The inclusion criteria were as follows: aged between 18 and 90 years old, diagnosed with ESRD, and under regular dialysis for more than 3 months. The exclusion criteria were as follows: the presence of active bleeding due to major trauma, gastric ulcer, or surgery, a recent history of blood transfusion, and/or evidence of malignancy or uncontrolled hypertension. In addition, patients who required additional ESA treatments or whose CERA treatments were discontinued during the 27-week treatment regimen were withdrawn from the study.

Forty-five patients received an injection of 100 µg of Mircera once monthly for 2 months in phase 1. Then, they received an injection of 50 µg of Mircera twice monthly for 2 months in phase 2. Afterwards, they were shifted back to an injection of 100 µg of Mircera once monthly for another 2 months in phase 3.

Demographic and biochemical data were collected during the initiation of the ESA treatment in order to determine their association with ESA effectiveness under different dosage intervals. The demographic data collected included age, gender, body weight, dialysis duration, diabetes and hepatitis status, and dialysis adequacy index (Kt/V, determined by Daugirdas method). The laboratory data included the following:

(1) Complete blood count: white blood cell (WBC) count, red blood cell (RBC) count, hemoglobin (HB), hematocrit (HCT), mean corpuscular volume (MCV), mean corpuscular hemoglobin (MCH), mean corpuscular hemoglobin concentration (MCHC), red blood cell distribution width (RDW), and platelet (PLT) count;

(2) Biochemical profile: total protein (TP), albumin (Alb), total cholesterol (CHOL), triglyceride (TG), uric acid, glucose, blood urea nitrogen (BUN), creatinine (Cr), sodium (Na), potassium (K), chloride (Cl), calcium (Ca), phosphate (P), total bilirubin (T.BILI), alkaline phosphatase (ALK-P), γ-glutamyl transferase (GGT), alanine aminotransferase (ALT), aspartate aminotransferase (AST), creatine kinase (CK), and lactate dehydrogenase (LDH);

(3) Storage iron status: serum iron, total iron-binding capacity (TIBC), transferrin saturation (TSAT), ferritin; 

(4) Inflammation markers: c-reactive protein (CRP), tumor necrosis factor (TNF), interleukin-1 (IL-1), interleukin-6 (IL-6), and hepcidin. These markers were checked in the middle of phase 1 and phase 3.

A good (erythropoietin) response is defined as a 1% increase in HCT level (e.g., from 30% at baseline to 31% after intervention) [16]. There were 17 patients who qualified as good responders (GR), while there were 28 patients who were poor responders (PR). The demographic and biochemical factors were compared between GR and PR groups to identify significant factors that influenced erythropoietic response, which was determined by the change in HCT level from phase 1 to phase 3 (Δ HCT). A previous study had shown that hematocrit levels, inflammation, and nutrition status all improved during a 2-month period of more frequent ESA injections [17].

### 2.2. Measurement of PFOA and PFOS

Serum PFC (PFOA and PFOS) levels were measured using liquid chromatography-tandem mass spectrometry (LC-MS/MS) with isotope dilution in this study. The main particles used in LC-MS/MS to quantify PFCs were serum PFOA (*m/z* 413→369) and PFOS (*m/z* 499→80). The detailed analytical procedure and product ion scan of the PFCs are described in the Appendix A.

### 2.3. Statistical Analysis

The patient sample size was determined based on an effective size to identify the significance of differences in Δ HCT among different groups. If a 5% chance of a type I error (α = 0.05) was permitted, with a power of 90%, assuming the difference of Δ HCT among the two different groups was at least equal to the standard derivation, then an adequate sample size would be 42 patients. Therefore, a total of 45 patients were enrolled in this study.

Statistical analysis was performed using SPSS 19.0. Continuous data were expressed as mean ± standard deviation. *T*-test or ANOVA were conducted to analyze the categorical variables with erythropoietic response. A linear regression was performed to analyze the association of demographic and biochemical factors with erythropoietic response. Factors that showed significant association with decreased Δ HCT were defined as poor erythropoietic factors. Patients were divided into subgroups based on the different numbers of poor erythropoietic factors, and the Δ HCT levels were compared. A *p* value of < 0.05 was considered statistically significant.

## 3. Results

**Figure 1:**
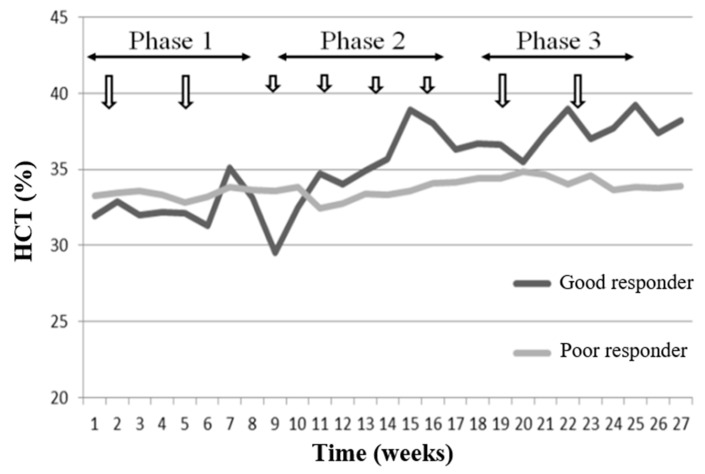
Mean HCT levels of GR and PR during the 27-week study (arrows indicate the timing of CERA injection, 100 µcg for phases 1 and 3 and 50 µcg for phase 2). Abbreviations: HCT, hematocrit; GR, good responder; PR, poor responder; CERA, continuous erythropoietin receptor activator.

The mean HCT levels of GR and PR groups during the study period are illustrated in Figure 1. The GR group had a lower HCT level in phase 1 (32.90 ± 2.25), but had a higher HCT level (35.71 ± 1.81) in phase 3. In contrast, the HCT levels in the PR group during phases 1 and 3 were approximately the same, at 33.77 ± 3.16 and 33.45 ± 3.25, respectively. Thus, the erythropoietic response in the GR group was more sensitive to the CERA injection than the PR group.

Next, the demographic and biochemical factors were analyzed in association with erythropoietic response (Δ HCT) (Table 1). Higher glucose levels (*p* = 0.008), lower LDH levels (*p* = 0.046), higher ferritin levels (*p* = 0.045), and higher IL-6 levels (*p* = 0.016) were associated with a poor erythropoietic response. Linear regression analysis was performed to further analyze the association of factors with Δ HCT and revealed additional significant factors associated with a poor erythropoietic response, such as older age (*p* = 0.026), higher initial HCT levels (*p* = 0.049), lower Cl levels (*p* = 0.025), higher ALT levels (*p* = 0.034), and higher CRP levels (*p* = 0.001), in addition to the confirmation of the results of the previous analysis: glucose (*p* = 0.004), LDH (*p* = 0.026), and IL-6 (*p* = 0.011), except for ferritin. There was no significant difference between the PFOA and PFOS levels of the GR and PR groups, and no significant association between the two types of PFCs with the erythropoietic response.

**Figure 2:**
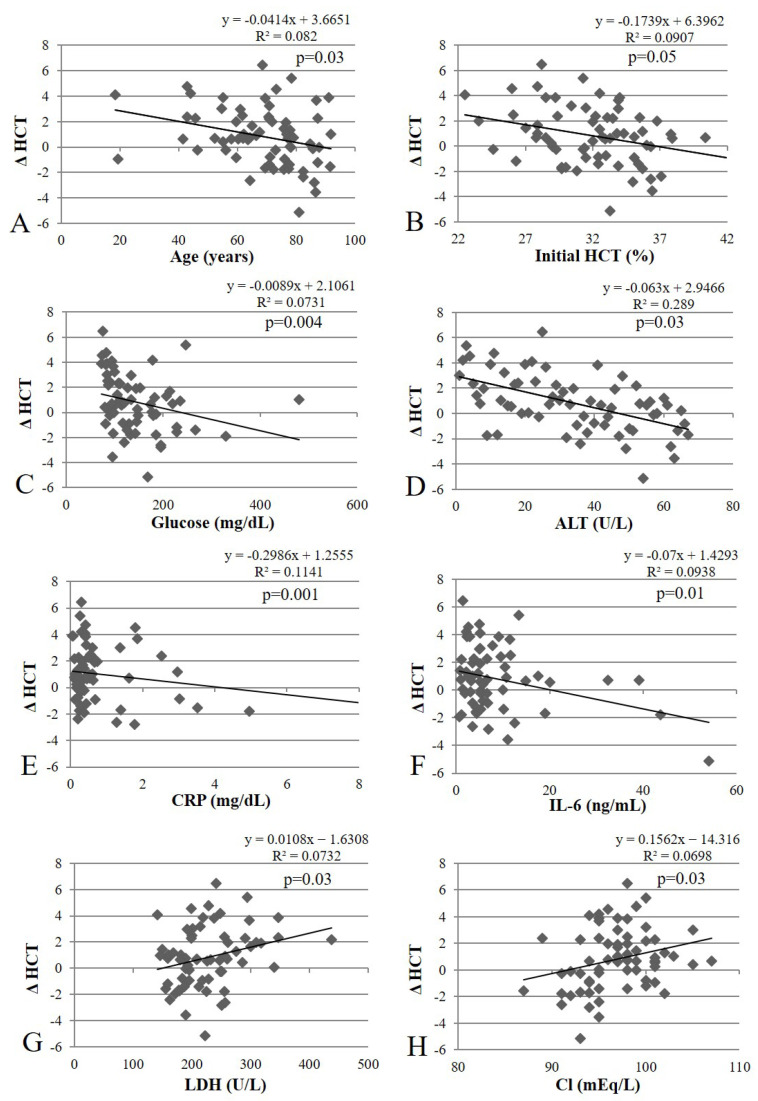
Linear regression analyses of significant factors associated with the erythropoietic response. Abbreviations: HCT, hematocrit; ALT, alanine aminotransferase; CRP, c-reactive protein; IL-6, interleukin-6; LDH, lactate dehydrogenase; Cl, chloride; Δ HCT, change in HCT level from phase 1 to phase 3.

The linear regression plots showing the significant factors associated with the erythropoietic response in Table 1 are displayed in Figure 2. Age, initial HCT, and levels of glucose, ALT, CRP, and IL-6 showed significant negative associations with the erythropoietic response, while LDH and Cl levels showed significant positive associations with the erythropoietic response.

**Figure 3:**
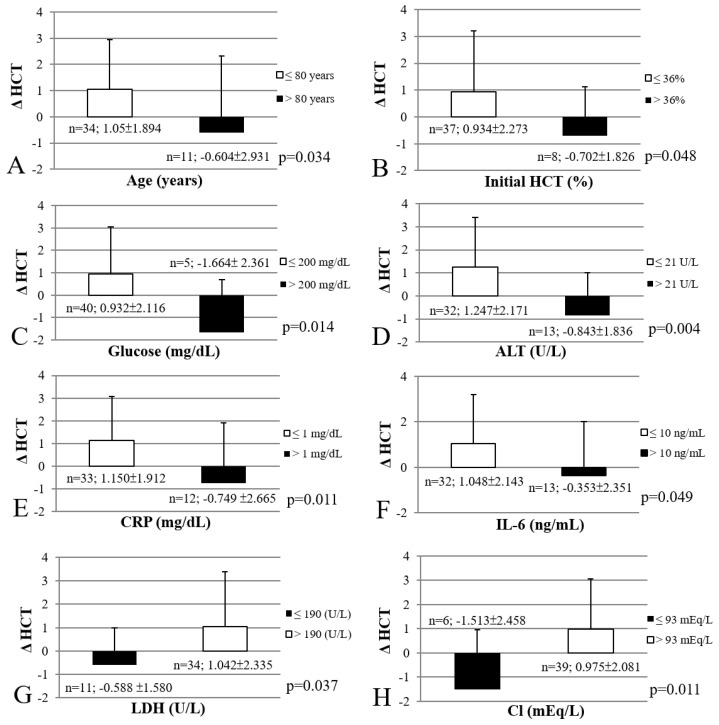
Subgroup analyses of significant factors associated with erythropoietic response. Abbreviations: HCT, hematocrit; ALT, alanine aminotransferase; CRP, c-reactive protein; IL-6, interleukin-6; LDH, lactate dehydrogenase; Cl, chloride; Δ HCT, change in HCT level from phase 1 to phase 3.

A subgroup analysis of the eight significant factors associated with erythropoietic response is shown in Figure 3. The cut-off values for each factor were as follows: age: 80 years old; initial HCT: 36%; glucose: 200 mg/dL; ALT: 21 U/L; CRP: 1 mg/dL; IL-6: 10 ng/mL; LDH 190 U/L; Cl: 93 mEq/L. The subgroup of patients who exceeded the cut-off values for six factors exhibited significantly poorer erythropoietic responses: age above 80 years (*p* = 0.034), initial HCT levels higher than 36% (*p* = 0.048), glucose levels higher than 200 mg/dL (*p* = 0.014), ALT levels higher than 21 U/L (*p* = 0.004), CRP levels higher than 1 mg/dL (*p* = 0.011), and IL-6 levels higher than 10 ng/mL (*p* = 0.049). However, patients with LDH and Cl levels below the designated cut-off values had significantly poorer erythropoietic responses (*p* = 0.037, *p* = 0.011, respectively).

Patients were further grouped according to the number of poor erythropoietic factors they exhibited (group 1: no poor erythropoietic response factor, group 2: one poor erythropoietic response factor, group 3: two poor erythropoietic response factors, and group 4: three or more poor erythropoietic response factors), and the analysis of their erythropoietic responses is shown in Table 2. Group 1 demonstrated a significant increase in HCT levels (2.3%), followed by group 2 (1.2%). It was evident that as the number of poor erythropoietic response factors increased, the magnitude of the increase in HCT levels decreased, indicating a poor erythropoietic response. Group 3 only had a 0.1% increase in HCT levels, whereas group 4 had a 1.3% decrease in HCT levels. This suggests that patients with less than two poor erythropoietic response factors may benefit from a strategy of increased frequency of a fixed CERA dosage.

The multivariate linear regression model of factors associated with erythropoietic response is shown in Table 3, where only IL-6 reached statistical significance (*p* = 0.034). Inflammation markers, such as CRP, TNF-α, IL-1, IL-6, and hepcidin, were then further analyzed for association with PFCs in Table 4. IL-1 was positively associated with PFOA (*p* = 0.088), while IL-6 was positively associated with PFOS (*p* = 0.068), although these values only reached borderline significance.

## 4. Discussion

**Figure 4:**
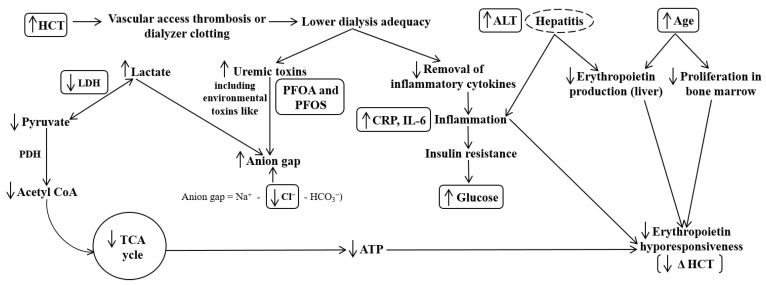
Schematic representation of possible mechanisms involving significant factors associated with erythropoietic response. Abbreviations: HCT, hematocrit; ALT, alanine aminotransferase; LDH, lactate dehydrogenase; PDH, pyruvate dehydrogenase; acetyl CoA, acetyl coenzyme A; TCA cycle, tricarboxylic acid cycle; ATP, adenosine triphosphate; PFOA, perfluorooctanoic acid; PFOS, perfluorooctane sulfonate; Na^+^, sodium; Cl^−^, chloride; HCO_3_^−^, bicarbonate; CRP, c-reactive protein; IL-6, interleukin-6; Δ HCT, change in HCT level from phase 1 to phase 3.

After changing the dosage and frequency of the CERA administration from 100 µg once monthly to 50 µg twice monthly, eight factors were found to be significantly associated with a poor erythropoietic response, namely age, initial HCT level, as well as glucose, ALT, CRP, IL-6, LDH, and Cl levels; the mechanisms of these associations are illustrated in Figure 4.

Age was a significant predictor of erythropoietic response. Compared to a previous study where 65 years was defined as elderly [18], the majority of the patients enrolled in this study were older (69 ± 14 years old). Therefore, an age of 80 years was set as the cut-off value; 80 years is also the approximate mean life expectancy of people in Taiwan, where the study is conducted [19]. It was found that the benefit of higher dosing frequency of CERA decreases with age, since the erythropoietic response of patients older than 80 years old was worse than those 80 years old and under. This may be explained by age-related bone marrow inflammation and/or the presence of multiple comorbidities in older people. An aging bone marrow microenvironment plays a critical role in the development of ineffective erythropoiesis [20].

The initial HCT level was the only component of the complete blood count, showing a significant association with the erythropoietic response. According to the CHOIR (Correction of Hemoglobin and Outcomes in Renal Disease) and CREATE (Cardiovascular Risk Reduction by Early Anemia Treatment with Epoetin Beta) trials, the target range of HCT in uremic patients under HD is 30–36%, which is approximately equivalent to HB levels of 10–12 g/dL [21]. Implementing the optimal HB target range led to a significantly longer survival in patients with chronic kidney disease (CKD) who died during the period of 2007–2009, than in those who died during 2000–2006 in a 2013 study analyzing Australia’s Renal Anemia Database. However, HB levels above 12 g/dL were linked to an increased risk of cardiovascular (CV) complications and death [22]. HCT levels above 12 g/dL may be associated with hypertension, vascular access thrombosis, and/or dialyzer clotting [16,21]. These complications may lead to lower dialysis adequacy (patients in the PR group have slightly lower Kt/V values (1.62 ± 0.21) than patients in the GR group (1.71 ± 0.17), which, in turn, may lead to a poor erythropoietic response. This is consistent with the findings in this study where higher initial HCT levels were associated with a poor erythropoietic response. Since such patients already possess upper limit HCT values with a CERA dosage of 100 µg, there is little to no room for HCT levels to increase without increasing the dosage.

Inflammation is a major factor determining the erythropoietic response. Among the inflammation markers analyzed, CRP and IL-6 showed significant negative associations with the erythropoietic response. Inflammation interferes with serum iron utilization and causes malnutrition, which eventually results in anemia [15,16,17,23]. CRP was associated with poor survival in CV events among the elderly [18]. Carotid intima-media thickness and CRP were independently associated with CV-event risk in patients with atherosclerotic occlusive disease [24]. In a 2011 study focused on protein-energy wasting and inflammation, malnutrition was associated with all-cause mortality and CV events, while CRP levels were higher in hyporesponders and predicted all-cause mortality and CV events. Multiple studies showed that increased IL-6 levels were associated with increased ESA requirements due to decreased responsiveness to ESAs in HD patients [25,26,27]. Thus, these data are in agreement with the results of this study, where inflammation was found to be associated with a poor erythropoietic response.

Inflammation is commonly associated with glucose levels. Patients with higher glucose levels exhibited poorer erythropoietic responses. Proinflammatory cytokines can cause insulin resistance in various target tissues by inhibiting insulin signal transduction, leading to elevated glucose production [28].

According to the baseline data analysis, there were more patients in the PR group (25%) with hepatitis than in the GR group (5.9%). ALT is an indicator of liver function. Findings revealed that patients with higher ALT levels of > 21 U/L (even though the upper limit ALT values were considered below the normal range, patients with renal failure usually have low concentrations of ALT, approximately < 40 U/L [29,30]) were associated with a poor erythropoietic response. Erythropoietin production from non-kidney cells increases to compensate for insufficient renal erythropoietin production during CKD [31]. The liver may produce a certain amount of erythropoietin during renal failure; hence, liver dysfunction may be associated with impaired erythropoietin synthesis [31].

Inflammation may stem from lower dialysis adequacy where there is decreased clearance of proinflammatory cytokines, as well as the accumulation of uremic toxins [32,33]. Due to the retention of toxins, many of which are anionic metabolites, such as lactate, ketone, phosphate, and sulfate, the anion gap increases, especially in the later stages of CKD [34]. Serum anion gap is calculated by the following formula: serum anion gap = Na^+^–Cl^−^–HCO_3_^−^. Thus, the formula indicates that decreased Cl levels may lead to a high anion gap. This is in agreement with the results of this study, where patients with lower Cl levels showed a poor erythropoietic response.

In this study, patients with lower LDH levels were associated with poor erythropoietic responses. Lower LDH levels may cause an inadequate conversion of lactate (one of the accumulated anionic toxins that may contribute to a high anion gap during CKD) to pyruvate, which in turn leads to reduced acetyl coenzyme A levels, affecting the tricarboxylic acid cycle and resulting in reduced adenosine triphosphate levels. The reduced energy state may interfere with the erythropoietic response. Higher LDH levels may indicate higher erythropoietic activity [35]. There was a similar finding in a 2016 study, where serum LDH levels and RBC count increased upon changing the ESA treatment from darbepoetin-alpha to CERA in uremic patients. LDH was also considered to be a marker since a higher LDH level indicates a higher ESA response. Chloride may be an indicator for dehydration in patients under hemodialysis since sodium is clamped during the process [15].

There were no significant differences between the PFOA and PFOS levels of the GR and PR groups and between the two types of PFCs with erythropoietic response. There was also only a borderline-significant association between inflammation and PFCs. One explanation may be that PFCs reflect the long-term physical condition of uremic patients [23], while the erythropoietic response is more influenced by acute inflammation. The half-life of PFCs is longer than 6 months and may be too long to coincide with acute inflammation [16]. Another possibility could be that the PFC levels measured were too low to reach statistical significance in influencing the erythropoietic response. Adequate dialysis may have lowered the PFC levels in patients [36]. The patients enrolled in this study exhibited the recommended Kt/V of at least 1.2. This may be why no significant association was noted between PFCs and the erythropoietic response. Moreover, it is difficult to show the adverse effect of PFCs on erythropoiesis because of the difficulty to enroll a study population that will reach an effective size to determine the effects of these environmental toxins. Uremic patients are at a risk of developing other diseases and may require blood transfusion. Patients with these conditions would typically be excluded from the study [17]. In future research, patients who are not undergoing dialysis can also be included in order to examine the influence of dialysis on PFCs. The study can also be replicated in a different region with higher toxin background levels, such as an industrial region, to confirm the effects seen in this study [23].

To our knowledge, this is the first study conducted to investigate the influence of PFCs on the erythropoietic response of uremic patients under HD. The findings may provide meaningful information for future studies that should enroll more patients to understand how PFCs act on CKD patients. This study presents the personalization and prediction of the erythropoietic response of HD patients by offering the option of increased CERA frequency to physicians when treating anemia in a clinical setting.

## 5. Conclusions

The evaluation of a fixed CERA dosage administered at an increased frequency to HD patients indicated that certain factors such as age, glucose, Cl, liver function, and inflammation, may be associated with a poor erythropoietic response. Study findings provide evidence that may be applied in clinical practice to determine the cost-effectiveness of ESA administration.

## Figures and Tables

**Table 1:** Baseline demographic and biochemical factors associated with erythropoietic response.

Factors	CERA Initiation *N* = 45	GR *N* = 17	PR *N* = 28	*p*	Linear Regression Coefficient	*p*
Male	26 (57.77%)	9 (52.94%)	17 (60.71%)	0.757	N/A	N/A
DM	23 (51%)	10 (58.8%)	13 (46.4%)	0.341	N/A	N/A
Hepatitis	8 (17.77%)	1 (5.88%)	7 (25%)	0.132	N/A	N/A
Age (years)	69.44 ± 13.66	67.33 ± 14.65	70.84 ± 13.37	0.415	−0.055	0.026 *
HD duration (years)	9.23 ± 16.52	7.60 ± 4.53	10.51 ± 4.71	0.576	0.010	0.638
BW (kg)	59.32 ± 11.86	57.01 ± 14.30	60.08 ± 10.26	0.285	−0.038	0.185
URR (%)	0.73 ± 0.10	0.74 ± 0.05	0.70 ± 0.11	0.204	0.882	0.805
Kt/V	1.67 ± 0.61	1.71 ± 0.17	1.62 ± 0.21	0.139	0.503	0.673
WBC (10^3^/mm^3^)	6.83 ± 1.54	6.58 ± 1.49	6.91 ± 1.58	0.497	0	0.705
RBC (10^6^/mm^3^)	3.51 ± 0.47	3.39 ± 0.30	3.58 ± 0.54	0.204	−0.838	0.254
HB (g/dl)	11.10 ± 1.13	10.87 ± 0.92	11.26 ± 1.23	0.272	−0.539	0.074
HCT (%)	33.30 ± 3.44	32.56 ± 2.68	33.81 ± 3.83	0.245	−0.167	0.049 *
MCV (fl)	95.51 ± 6.58	95.98 ± 3.56	95.18 ± 8.01	0.699	−0.034	0.517
MCH (pg)	31.86 ± 2.55	32.02 ± 1.42	31.74 ± 3.09	0.725	−0.096	0.476
MCHC (g/dL)	33.34 ± 0.68	33.37 ± 0.64	33.32 ± 0.72	0.832	−0.262	0.605
RDW (%)	15.90 ± 1.59	15.96 ± 1.53	15.83 ± 1.67	0.801	0.043	0.866
PLT (10^5^/mm^3^)	1.79 ± 0.58	1.89 ± 0.50	1.73 ± 0.62	0.395	0	0.383
TP (g/dL)	6.92 ± 0.66	6.92 ± 0.55	6.92 ± 0.71	0.983	−0.332	0.536
Alb (g/dL)	4.03 ± 0.39	3.98 ± 0.37	4.06 ± 0.41	0.559.	−0.197	0.822
CHOL (mg/dL)	172.07 ± 43.69	174.41 ± 39.32	170.82 ± 46.79	0.783	0	0.961
TG (mg/dL)	199.53 ± 175.31	173.00 ± 103.57	215.64 ± 207.39	0.435	−0.002	0.441
UA (mg/dL)	7.05 ± 1.63	7.33 ± 1.54	6.88 ± 1.68	0.379	0.073	0.732
Glucose (mg/dL)	118.26 ± 48.98	96.82 ± 23.44	130.68 ± 56.68	0.008 *	−0.019	0.004 *
BUN (mg/dL)	68.41 ± 20.32	66.29 ± 21.75	68.21 ± 18.46	0.753	−0.014	0.436
CREA (mg/dL)	10.29 ± 2.61	10.82 ± 3.11	9.99 ± 2.31	0.317	0.066	0.615
Na (mEq/L)	137.48 ± 3.50	137.76 ± 2.19	137.50 ± 4.05	0.806	0.144	0.150
K (mEq/L)	4.85 ± 0.70	4.78 ± 0.61	4.84 ± 0.74	0.778	−0.089	0.861
Cl (mEq/L)	97.46 ± 4.04	98.41 ± 3.12	97.11 ± 4.39	0.291	0.191	0.025 *
Ca (mg/dL)	9.50 ± 0.84	9.54 ± 0.73	9.46 ± 0.92	0.775	0.032	0.939
P (mg/dL)	5.28 ± 1.58	5.39 ± 1.81	5.19 ± 1.48	0.688	0.259	0.230
T.BILI (mg/dL)	0.25 ± 0.12	0.25 ± 0.11	0.26 ± 0.13	0.791	−1.629	0.580
ALK-P (U/L)	90.98 ± 44.57	97.53 ± 54.86	86.85 ± 37.25	0.446	0.005	0.530
GGT (U/L)	30.02 ± 27.78	29.53 ± 29.59	30.33 ± 27.14	0.927	−0.015	0.226
ALT (U/L)	21.08 ± 24.70	15.76 ± 6.03	19.59 ± 8.59	0.091	−0.093	0.034 *
AST (U/L)	18.42 ± 8.05	19.18 ± 7.07	19.59 ± 7.80	0.859	−0.035	0.460
CK (U/L)	58.20 ± 28.62	61.12 ± 30.72	56.43 ± 27.68	0.6	0.022	0.069
LDH (U/L)	231 ± 54.45	251.71 ± 65.85	218.43 ± 42.74	0.046 *	0.011	0.026 *
Iron (μg/dL)	68.86 ± 26.26	60.12 ± 24.90	74.37 ± 26.19	0.081	−0.011	0.409
TIBC (μg/dL)	215.50 ± 35.55	215.71 ± 34.47	215.37 ± 36.86	0.976	−0.007	0.499
TSAT (%)	0.33 ± 0.14	0.31 ± 0.17	0.34 ± 0.13	0.522	0.824	0.737
Ferritin (ng/mL)	500.81 ± 373.77	353.31 ± 33.81	585.49 ± 379.86	0.045 *	−0.001	0.119
CRP (mg/dL)	0.96 ± 1.70	0.634 ± 0.759	1.201 ± 2.1	0.294	−0.626	0.001 *
TNF-α (ng/mL)	37.72 ± 15.01	38.65 ± 17.54	36.44 ± 13.35	0.650	−0.023	0.368
IL-1 (ng/mL)	10.77 ± 21.99	9.18 ± 17.19	12.22 ± 25.16	0.675	−0.007	0.677
IL-6 (ng/mL)	9.53 ± 11.72	4.91 ± 3	12.50 ± 14.34	0.016 *	−0.078	0.011 *
Hepcidin (ng/mL)	84.58 ± 42.62	67.45 ± 44.79	93.40 ± 38.12	0.054	0.004	0.749
PFOA (ng/mL)	0.19 ± 0.02	0.20 ± 0.04	0.184 ± 0.01	0.151	24.263	0.109
PFOS (ng/mL)	0.17 ± 0.19	0.120 ± 0.05	0.195 ± 0.228	0.201	−0.360	0.856

Categorical variables are expressed in number and percentage (N (%)), while continuous variables are expressed in mean and standard deviation (mean ± SD). Variables with significant associations are indicated with asterisks at significance level of *p* < 0.05. Abbreviations: CERA continuous erythropoietin receptor activator; GR, good responder; PR, poor responder; HCT, hematocrit; DM, diabetes mellitus; HD, hemodialysis; BW, body weight; URR, urea reduction ratio; Kt/V, dialysis efficiency; WBC, white blood cell count; RBC, red blood cell count; HB, hemoglobin; MCV, mean corpuscular volume; MCH, mean corpuscular hemoglobin; MCHC, mean corpuscular hemoglobin concentration; RDW, red blood cell distribution width; PLT, platelet count; TP, total protein; Alb, albumin; CHOL, total cholesterol; TG, triglyceride; UA, uric acid; BUN, blood urea nitrogen; CREA, creatinine; Na, sodium; K, potassium; Cl, chloride; Ca, calcium; P, phosphate; T.BILI, total bilirubin; ALK-P, alkaline phosphatase; GGT, γ-glutamyltransferase; ALT, alanine aminotransferase; AST, aspartate transaminase; CK, creatine kinase; LDH, lactate dehydrogenase; TIBC, total iron-binding capacity; TSAT, transferrin saturation; CRP, c-reactive protein; TNF-α, tumor necrosis factor-α; IL-1, interleukin-1; IL-6, interleukin-6; PFOA, perfluorooctanoic acid; PFOS, perfluorooctane sulfonate; N/A, not available.

**Table 2:** Patients grouped according to the number of poor erythropoietic response factors associated with erythropoietic response.

Group	Number of Patients	Δ HCT (mean ± SD)	*p* (Reference: Group 3)
1	12	2.27 ± 2.12	0.001 *
2	11	1.24 ± 1.59	0.035 *
3	12	0.11 ± 1.36	0.39
4	10	−1.32 ± 2.45	

Note: group 1: no poor erythropoietic response factor, group 2: one poor erythropoietic response factor, group 3: two poor erythropoietic response factors, and group 4: ≥3 poor erythropoietic response factors (consisted of: four persons with three poor erythropoietic response factors, two persons with four poor erythropoietic response factors, and four persons with five poor erythropoietic response factors). Variables with significant associations are indicated with asterisks at significance level of *p* < 0.05. Abbreviation: Δ HCT, change in HCT level from phase 1 to phase 3.

**Table 3:** Multivariate linear regression model of factors associated with erythropoietic response.

Factors Associated with Δ HCT	Linear Regression Coefficient	*p*
Age (for each 1 year increase)	−0.038	0.135
Initial HCT (for each 1% increase)	−0.151	0.119
ALT (for each 1 U/L increase)	−0.051	0.295
LDH (for each 1 U/L increase)	0.008	0.163
Cl (for each 1 mEq/L increase)	0.068	0.452
IL-6 (for each 1 ng/mL increase)	−0.059	0.034 *

Abbreviations: Δ HCT, change in HCT level from phase 1 to phase 3; HCT, hematocrit; ALT, alanine aminotransferase; LDH, lactate dehydrogenase; Cl, chloride; IL-6, interleukin-6. Variables with significant associations are indicated with asterisks at significance level of *p* < 0.05

**Table 4:** Linear regression of inflammation markers associated with PFCs.

Inflammation Markers	PFOA Linear Regression Coefficient	*p*	PFOS Linear Regression Coefficient	*p*
CRP (mg/dL)	−1.941	0.867	0.132	0.930
TNF-α (ng/mL)	−7.357	0.943	−19.856	0.121
IL-1 (ng/mL)	254.29	0.088	3.429	0.863
IL-6 (ng/mL)	−24.415	0.765	18.846	0.068
Hepcidin (ng/mL)	8.854	0.957	36.545	0.322

Abbreviations: PFCs, perfluorinated chemicals; PFOA, perfluorooctanoic acid; PFOS, perfluorooctane sulfonate; CRP, c-reactive protein; TNF-α, tumor necrosis factor-α; IL-1, interleukin-1; IL-6, interleukin-6.

**Figure A1:**
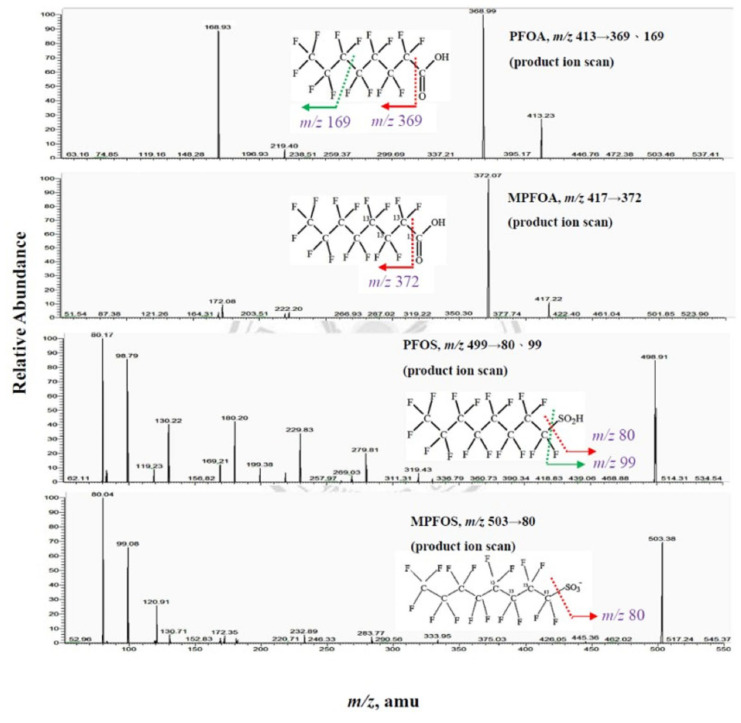
Product ion scan of PFOA and PFOS. Abbreviations: PFOA, perfluorooctanoic acid; MPFOA, monomethyl branch isomer of perfluorooctanoic acid; PFOS, perfluorooctane sulfonate; MPFOS, monomethyl branch isomer of perfluorooctane sulfonic acid.

## Data Availability

Data sharing not applicable.

## Appendix A

Measurement of perfluorooctanoic acid (PFOA) and perfluorooctane sulfonate (PFOS).

Serum samples of PFOA and PFOS were stored at −80 °C. The frozen serum samples were thawed at 4 °C and then vortex-mixed for 30 s to ensure homogeneity. A 50 µL serum sample was vortexed with 50 µL of 1% formic acid (pH 2.4) for 30 s. Forty microliters of acetonitrile and 1 µL of 10 µg/mL internal standard solution (^13^C_4_-PFOA and ^13^C_4_-PFOS, Wellington Laboratories Inc., Guelph, ON, Canada) were added to each sample before the second vortex. The sample was sonicated for 20 min and then centrifuged at 18,000× *g* for 20 min. The supernatant was collected and then filtered through a 0.22-µm polyether sulfone syringe filter into a vial.

The liquid chromatography-tandem mass spectrometry (LC-MS/MS) system used in this study consisted of the Agilent 1100 series system (Agilent Tech, Santa Clara, CA, USA) coupled to the Finnigan TSQ Quantum Discovery Max spectrometer system (Thermo Electron Corporation, Eindhoven, The Netherlands), with an electron spray ionization source in negative ion mode. PFOA and PFOS were quantified using LC-MS/MS coupled with isotope dilution. A 5 µL sample was injected onto a 2.0 mm × 150 mm Capcell Pak^®^ 3 µm C18 column (SHISEIDO Co, Tokyo, Japan) with mobile phases consisting of 10 mM ammonium acetate in water (A) and pure acetonitrile (B), delivered at a constant flow rate of 0.2 mL/min.

The mobile phase was kept at 30% B for 3 min after injection. A gradient was applied in 3 min to 65% B, then in 5 min to 100% B, where it was kept for 7 min. The column was then conditioned at 30% B for 1.5 min. The optimized MS parameters were as follows: spray ion voltage of 3000 V, capillary temperature of 210 °C, sheath gas pressure of 10 arbitrary units, auxiliary gas pressure of 5 arbitrary units, ion sweep gas pressure of 4 arbitrary units, collision gas pressure of 1.0 mTorr, and dwell time of 100 milliseconds. The detection was carried out in a selective reaction monitoring (SRM) mode. The collision energy (V) and selective reaction monitoring transitions monitored were as follows: 10 V, m/z 413→369 for PFOA; 12 V, m/z 417→372 for ^13^C_4_-PFOA; 40 V, m/z 499→80 for PFOS; 40 V, m/z 503→80 for ^13^C_4_-PFOS.

The quantification of PFOA and PFOS was performed by isotope dilution with stable isotope internal standards. The limit of quantification (LOQ) of PFOA and PFOS was 0.024 ng and 0.13 ng, respectively. The limit of detection (LOD) of PFOA and PFOS was 0.008 ng and 0.04 ng, respectively. The precision of PFOA and PFOS was at 13% and 8%, respectively, and the accuracy at 108% and 102%, respectively. For human blood samples, 10 calibration curve points were prepared at 0.25 ng/mL to 250 ng/mL standard. The recovery ranged at 88–105% for PFOA and PFOS.

The product ion scan of PFOA and PFOS is shown below.
